# Combined myoepithelial carcinoma and myoepithelioma in soft tissue: a case report and review of the literature

**DOI:** 10.1186/1752-1947-8-317

**Published:** 2014-09-24

**Authors:** Youssef Mahdi, Fouad Zouaidia, Abdelilah Zouhair, Mohamed Azouz, Kaoutar Znati, Ahmed Jahid, Mohamed Saleh Berrada, Zakiya Bernoussi, Fatima Mansouri, Moradh el Yaacoubi, Najat Mahassini

**Affiliations:** 1 Department of Pathology, Ibn Sina University Hospital, Rabat, Morocco; 2 Department of Orthopaedics and Traumatology, Ibn Sina University Hospital, Rabat, Morocco; 3 Faculty of Medicine and Pharmacy of the Mohammed V Souissi University, Rabat, Morocco

## Abstract

**Introduction:**

Soft tissue myoepithelial carcinoma and myoepithelioma are rare entities, part of myoepithelial tumors. They were incorporated into the World Health Organization classification of soft tissue tumors in 2002. Here we present an exceptional case of myoepithelial carcinoma and myoepithelioma association. To the best of our knowledge, such an association has never been reported in the literature.

**Case presentation:**

We report a case of myoepithelial carcinoma combined with myoepithelioma occurring in the soft tissue of the right forearm of an 84-year-old Arabian man. We describe the clinical, radiological and pathological features dominated by histological polymorphism. We will also describe the proposed histological criteria of malignancy and the major role of immunohistochemistry in positive and differential diagnosis. We finally mention the therapeutic arsenal available.

**Conclusion:**

Through this work, we report that myoepithelioma of soft tissue can progress to malignant myoepithelioma.

## Introduction

Soft tissue myoepithelial (ME) carcinoma, also known as soft tissue malignant myoepithelioma (STMM), and myoepithelioma are rare entities, part of ME tumors. They commonly occur in salivary glands, but they have also been reported in the nasopharynx, larynx, breast and lung [1,2]. Morphologically similar lesions arising primarily in soft tissue and bone have been described in the past 15 years [2]. They represent less than 1% of soft tissue tumors [3]. In 1995, Burke *et al*. reported the first primary myoepithelioma of soft tissue in retroperitoneal localization [4]. In 2002, the World Health Organization classification introduced ME tumors in soft tissue neoplasms. Hornick and Fletcher [5] published the largest series in 2003 with 101 cases, in which they proposed criteria for malignancy. Here, we present a case of ME carcinoma and myoepithelioma association. To the best of our knowledge, such an association in soft tissue has never been reported in the literature.

## Case presentation

**Figure 1:**
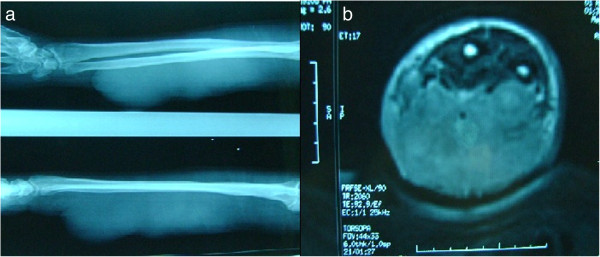
**Radiologic appearance of the tumor. a**: Arm X-rays show opaque lesion involving the entire forearm. It was located in the soft tissue without bone involvement. There was no calcification. **b**: Magnetic resonance imaging revealed an heterogeneous tumor process in the anterior face of the right forearm.

An 84-year-old Arabian man presented with a nodule of the anterior surface of his right forearm present for 30 years which had progressively increased in size. He benefited from resection of the nodule. Four months later, the mass recurred at the same location, gradually increased in volume and fistulated to his skin, causing a total loss of function of his right arm. A physical examination revealed a firm and deeply adhering mass of 20×10cm, which was painful with inflammatory signs. An X-ray of his arm showed no bone damage or soft tissue calcifications (Figure 1). Magnetic resonance imaging (MRI) revealed a heterogeneous tumor process in the anterior face of his right forearm measuring 13.7×5.5cm, isosignal at T1, slightly hypersignal at T2, including vascular structures (Figure 1). As a result, a surgical biopsy was performed.

**Figure 2:**
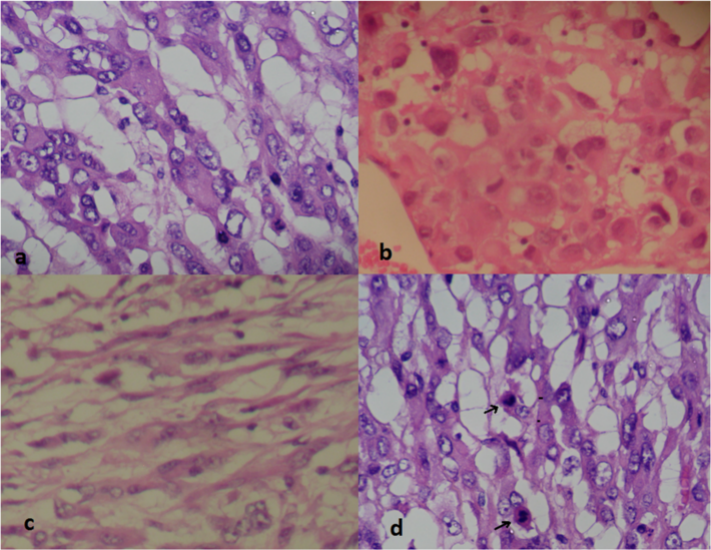
**Features of malignant myoepithelioma on histological examination (hematoxylin and eosin staining).** The neoplastic cells were epithelioid **(a)**, plasmacytoid **(b)**, and spindle **(c)**. They have frankly malignant cytomorphology with nuclear pleomorphism and vesicular chromatin **(d)**. Mitoses were numerous (d, arrows).

**Figure 3:**
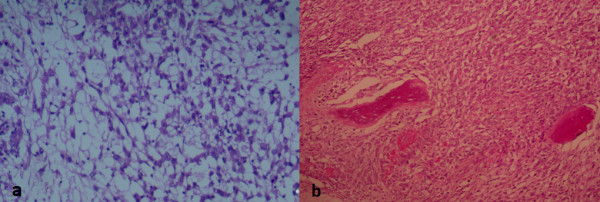
**Features of malignant myoepithelioma on histological examination (hematoxylin and eosin staining).** The matrix was myxoid **(a)**, with metaplastic bone **(b)**.

**Figure 4:**
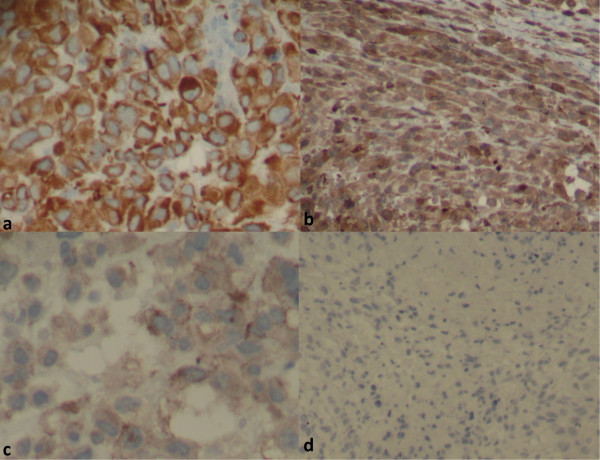
**Immunohistochemical profile of malignant myoepithelioma.** The neoplastic cells have epithelial and myoepithelial immunophenotype as well. They were positive for pancytokeratin (AE1/AE3) **(a)**, S-100 protein **(b)** and epithelial membrane antigen **(c)**. They were negative for desmin **(d)**, which eliminated diagnosis of rhabdomyosarcoma.

On microscopic examination, it was a malignant tumor proliferation, made of both cellular and loose areas. The neoplastic cells were epithelioid, plasmacytoid and spindle (Figure 2a, b and c). They have frankly malignant cytomorphology with nuclear pleomorphism and vesicular chromatin (Figure 2d). Mitoses were numerous (Figure 2d), estimated at 10 mitoses per 10 high-power fields. The matrix was myxoid, with metaplastic bone (Figure 3). Areas of necrosis were observed. Immunohistochemistry revealed positive staining with pancytokeratin, epithelial membrane antigen (EMA) and S-100 (Figure 4). The tumor was immunonegative for desmin, smooth muscle actin and CD34. A diagnosis of malignant myoepithelioma was established. *EWSR1* gene rearrangement research was non-contributory.

**Figure 5:**
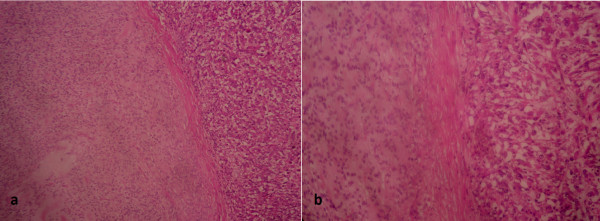
**Features of myoepithelioma on histological examination (hematoxylin and eosin staining).** Extensive sampling of the tumors reveals a very limited and encapsulated nodule **(a)**. It was highly cellular, demonstrating a solid growth pattern without stromal component. The neoplastic cells were only epithelioid. They show no cytological atypia **(b)**.

Given the locally advanced stage of the disease, an amputation of the limb was performed. An examination revealed the presence of a very limited and encapsulated nodule within the ME carcinoma. Neoplastic cells were epithelioid without cytological atypia, realizing myoepithelioma (Figure 5).

## Discussion

The histogenesis of soft tissue ME tumors is unknown, since normal ME cells are not found at these sites [2]. A few reports of genetic abnormalities have indicated *EWSR1* gene rearrangement in soft tissue ME tumors and, in one case each, the fusion partner was identified as either *PBX1* or *ZNF444*[6]. In a recent study of Antonescu *et al.*[6], 66 ME tumors mainly from soft tissue (71%), but also from skin, bone and visceral locations, characterized by classic morphological features and supporting immunoprofile were studied. The results do not support a pathogenetic relationship between soft tissue ME tumors and their salivary gland counterparts [6]. *EWSR1* gene rearrangement was a common event in ME tumors arising outside salivary glands, found in 45% of cases. *EWSR1-POU5F1* fusion was identified in a young patient, and in a subset of soft tissue ME tumors with clear cell morphology, which can be used as a molecular diagnostic test in difficult cases. *EWSR1-PBX1* fusion was found in a subset of ME tumors associated with a bland sclerotic appearance or clear cell morphology. *EWSR1-ZNF444* fusion was present in one case of ME tumors. *EWSR1*-negative tumors were more often benign, superficially located, and showed ductal differentiation. By contrast, one report describes a rare case of STMM without *EWSR1* gene rearrangement and an unusually poor outcome [7]: a 26-year-old man with STMM in his right hip. The tumor did not have an *EWSR1* gene rearrangement. It behaved aggressively and the patient died from multiple metastases 18 months after diagnosis. In another case of ME tumor in the pelvis of a 26-year-old man, considered as unresectable, *EWSR1-ATF1* fusion was identified, extending the spectrum of partner genes of *EWSR1*[8].

The age of patients in whom ME tumors present in soft tissue ranges from 3 to 83 years, with a mean of 40 years and a slight male predilection (1.4:1) [1,2,5]. Most patients present with a painful or painless mass [1]. The duration of symptoms ranges from 2 weeks to 20 years [1]. Nearly two-thirds of reported cases have arisen on the extremities (38% lower extremity and 27% upper extremity); the remainder has involved the head and the neck region (16%), trunk (13%) and visceral soft tissue (6%) [2]. Approximately 60% of tumors are subcutaneous in origin and 40% occur in deep soft tissue (intramuscular or subfascial) [2].

MRI is the radiological technique of choice [3]. In fact, it allows suspecting the diagnosis, and looking for a possible bone involvement in specifying the type (extension or pressure of the bone) [3].

The mean size for benign tumors is 4cm (range, 0.7 to 12cm) compared with 6cm (range, 1.3 to 20cm) for malignant lesions [2]. Most cases of soft tissue ME tumors are grossly well circumscribed [2,4], and may have a multinodular appearance [2].

On histological examination, the cells are most commonly arranged in trabecular or reticular patterns, but nested and solid areas are also frequently seen [2]. They can take on various morphological appearances, including spindled, epithelioid, plasmacytoid or clear cell features [2,5,9]. Typically, the epithelioid cells are present in combination with one or more other ME cell types [2]. The matrix is characteristically myxoid, chondromyxoid or hyalinized; metaplastic cartilage and/or bone are found in 15 to 20% of cases [2]. Squamous differentiation may be seen [9]. Of STMM, 40% show at least focal tumor necrosis [2]. According to the series of Hornick and Fletcher, infiltrative tumor margins and increased mitotic rates were frequently seen, but did not correlate to malignant behavior [5]. The only histological feature significantly associated with recurrence or metastasis was moderate or severe cytological atypia, which was defined by vesicular or coarse chromatin, prominent often large nucleoli, or nuclear pleomorphism [5]. On immunohistochemical examination, the neoplastic ME cells express the epithelial markers AE1/AE3 (90%) and EMA (60%); and they express the ME markers S-100 protein (89%), calponin (87%), glial fibrillary acidic protein (GFAP;46%), and smooth muscle actin (36%) [1].

Due to histological polymorphism, STMM poses significant challenges in differential diagnosis. This includes entities having architectural and cytological features similar to those of STMM, and sharing positivity of some antibodies in immunohistochemistry. This primarily involves epithelioid malignant peripheral nerve sheath tumor (MPNST), high-grade extraskeletal myxoid chondrosarcoma (EMC), epithelioid sarcoma, metastatic carcinoma and metastatic melanoma [2]. In addition to clinical data and histology, immunohistochemistry is often necessary. High-grade EMC are uniformly negative for keratin, and GFAP expression is rare [2]. Epithelioid MPNST generally do not express epithelial markers [2]. In addition, many epithelioid MPNSTs show foci of conventional spindle cell MPNST and a nerve of origin or a pre-existing benign nerve sheath tumor can be identified [2]. For proximal-type epithelioid sarcoma, S-100 expression is rare and GFAP is negative [2]. In sclerosing epithelioid fibrosarcoma, keratin and S-100 are usually negative [2]. The absence of S-100 and GFAP expression and the lack of epithelial markers allow exclusion of metastatic carcinoma or melanoma respectively [2]. The use of a complete immunohistochemical panel is critical. In our case, it was key to the positive diagnosis. In fact, if the antibody anti-S-100 protein was not done, we would not have made the diagnosis of ME carcinoma on biopsy.

Complete surgical resection with negative margins is the recommended treatment [1,2]. For metastatic STMM, experience with chemotherapy and radiation therapy is limited. The first reported case of a complete pathological response was a 37-year-old woman with metastatic STMM of the vulva in 2006 [10]. She received paclitaxel-carboplatin combination chemotherapy with a complete pathological response, and no further recurrence for more than 3 years.

## Conclusions

In our case, we had a tumor of soft parts which was locally invasive. Diagnosis of STMM was established in view of a wide variation in cellular morphology, severe cytological atypia, and epithelial and ME immunophenotype. It developed on myoepithelioma after the first surgery. Through this work, we report that myoepithelioma of soft tissue can progress to malignant myoepithelioma.

## Consent

Written informed consent was obtained from the patient for publication of this case report and accompanying images. A copy of the written consent is available for review by the Editor-in-Chief of this journal.

## Abbreviations

EMA: Epithelial membrane antigen; EMC: Extraskeletal myxoid chondrosarcoma; GFAP: Glial fibrillary acidic protein; ME: Myoepithelial; MPNST: Malignant peripheral nerve sheath tumor; MRI: Magnetic resonance imaging; STMM: Soft tissue malignant myoepithelioma.

## Competing interests

The authors declare that they have no competing interests.

## Authors’ contributions

YM analyzed and interpreted the patient data, drafted the manuscript and made the figures. FZ performed the histological examination, made the diagnosis, supervised YM and revised the manuscript. KZ, AJ, ZB, FM, MSB and ME provided valuable insight during manuscript preparation. AZ and MA participated in the analysis and interpretation of patient data. NM supervised the entire case. All authors read and approved the final manuscript.
